# Quantification of Movement-Related EEG Correlates Associated with Motor Training: A Study on Movement-Related Cortical Potentials and Sensorimotor Rhythms

**DOI:** 10.3389/fnhum.2017.00604

**Published:** 2017-12-11

**Authors:** Mads Jochumsen, Cecilie Rovsing, Helene Rovsing, Sylvain Cremoux, Nada Signal, Kathryn Allen, Denise Taylor, Imran K. Niazi

**Affiliations:** ^1^SMI, Department of Health Science and Technology, Aalborg University, Aalborg, Denmark; ^2^LAMIH, UMR Centre National de la Recherche Scientifique 8201, Université de Valenciennes et du Hainaut-Cambrésis, Valenciennes, France; ^3^Health and Rehabilitation Research Institute, Auckland University of Technology, Auckland, New Zealand; ^4^Center for Chiropractic Research, New Zealand College of Chiropractic, Auckland, New Zealand

**Keywords:** movement-related cortical potentials, EEG, grasp, motor training, ERD/ERS

## Abstract

The ability to learn motor tasks is important in both healthy and pathological conditions. Measurement tools commonly used to quantify the neurophysiological changes associated with motor training such as transcranial magnetic stimulation and functional magnetic resonance imaging pose some challenges, including safety concerns, utility, and cost. EEG offers an attractive alternative as a quantification tool. Different EEG phenomena, movement-related cortical potentials (MRCPs) and sensorimotor rhythms (event-related desynchronization—ERD, and event-related synchronization—ERS), have been shown to change with motor training, but conflicting results have been reported. The aim of this study was to investigate how the EEG correlates (MRCP and ERD/ERS) from the motor cortex are modulated by short (single session in 14 subjects) and long (six sessions in 18 subjects) motor training. Ninety palmar grasps were performed before and after 1 × 45 (or 6 × 45) min of motor training with the non-dominant hand (laparoscopic surgery simulation). Four channels of EEG were recorded continuously during the experiments. The MRCP and ERD/ERS from the alpha/mu and beta bands were calculated and compared before and after the training. An increase in the MRCP amplitude was observed after a single session of training, and a decrease was observed after six sessions. For the ERD/ERS analysis, a significant change was observed only after the single training session in the beta ERD. In conclusion, the MRCP and ERD change as a result of motor training, but they are subject to a marked intra- and inter-subject variability.

## Introduction

Acquiring motor skills is important throughout the lifespan, learning to ride a bike, learning new fine motor skills with the hands or relearning movements that are lost due to acquired brain injury such as stroke. Depending on the motor skill a large amount of motor training is needed for motor learning. Neural plasticity is believed to underlie the behavioral changes associated with motor learning during motor training (Pascual-Leone et al., 1995, 2005). Changes in neural plasticity have been reported in numerous studies of motor skill leaning (Pascual-Leone et al., 1995, 2005), following stroke and during post-stroke motor rehabilitation (Cramer, 2008a,b; Dimyan and Cohen, 2011). These changes include altered cortical maps and changes in the excitability of the neural pathways in the brain. While motor training outcomes can be relatively easily quantified at a behavioral level, using such measures as task completion time, changes in neural plasticity are more difficult to quantify. Different techniques exist to quantify neural plasticity including; functional magnetic resonance imaging (fMRI), magnetoencephalography (MEG), electroencephalography (EEG), and transcranial magnetic stimulation (TMS) (Auriat et al., 2015). Imaging techniques, such as fMRI provide information about the size of different brain structures and neural generators, but these techniques are expensive and technically demanding. TMS provides information about the cortical excitability and the size of cortical motor areas and is often used to quantify changes in neural plasticity associated with motor training in healthy subjects and people with stroke (Pascual-Leone et al., 1995; Mrachacz-Kersting et al., 2016). However, there are a number of contraindications to the use of TMS that leads to the exclusion of many participants in research studies (Rossi et al., 2009). It would be preferable if the changes in neural plasticity associated with motor training and learning could be quantified using a simple, cost-effective technique. Recently, there has been a reduction in the cost of EEG systems and considerable progress in the recording devices and signal processing techniques used. Different EEG derived measures have been shown to change after motor learning including EEG coherence (Andres et al., 1999; Gerloff et al., 2006; Mehrkanoon et al., 2016), source localization (Schwenkreis et al., 2007), and changes in EEG rhythms (Haufler et al., 2000; Finnigan and van Putten, 2013; Mak et al., 2013; Cooke et al., 2014). We wanted to investigate if other EEG derived measures, namely the movement-related cortical potential (MRCPs) and event-related synchronization/desynchronization could be used to measure changes neural plasticity with motor learning (Hatta et al., 2009; Masaki and Sommer, 2012; Aono et al., 2013).

The MRCP includes two distinct event-related potentials; the Bereitschaftspotential and Contingent Negative Variation (Hatta et al., 2009; Masaki and Sommer, 2012; Yilmaz et al., 2015). The former is associated with a self-paced movement, while the latter is associated with a cue-based movement. The MRCP is divided into different phases. The early part of the initial negative phase of the MRCP starts up to 2 s prior the movement onset, and it is seen as a slight increase in negativity (in this paper we define this as the readiness potential). This phase is interpreted as movement preparation and the primary neural generators are the supplementary motor area, pre-motor cortex and the cingulate motor areas (Shibasaki and Hallett, 2006). Approximately 0.5 s before the movement onset the late part of the initial negative phase starts (defined as the negative slope). This is seen as a steeper increase in negativity and it is generated with an increased contribution from the primary motor cortex (Shibasaki and Hallett, 2006). The maximum amplitude of the MRCP is seen around the movement onset which is produced primarily by the contralateral (to the movement) primary motor cortex (defined as the motor potential) (Shibasaki and Hallett, 2006). A number of studies have reported the effect of induction of plasticity, motor training or skill acquisition on the amplitude of the MRCP segments with conflicting results. Increased amplitudes have both been associated with increased and decreased cortical excitability (Birbaumer et al., 1990; Rossi et al., 2000; Holler et al., 2006; Lu et al., 2009, 2011; de Tommaso et al., 2012; Thacker et al., 2014; Sato et al., 2015), which is also associated with motor skill acquisition (Pascual-Leone et al., 1995). In studies investigating motor skill training specifically, the MRCP amplitude has been reported to increase and decrease as well (Taylor, 1978; Lang et al., 1983, 1986; Niemann et al., 1991; Staines et al., 2002; Chiang et al., 2004; Smith and Staines, 2006, 2010, 2012; Wright et al., 2012a). In general, the single-session studies could be considered as motor training. Most of these studies show an increase in MRCP amplitude as a result of a single training session (Taylor, 1978; Lang et al., 1983, 1986; Staines et al., 2002; Chiang et al., 2004; Smith and Staines, 2006, 2010, 2012; Wright et al., 2012a); however, a reduction in MRCP amplitude has also been observed (Niemann et al., 1991). For studies, where a task is trained over several weeks, MRCP amplitudes have been shown to both decrease (Wright et al., 2012a) and increase (Chiang et al., 2004). Long-term motor training over several years have been studied with professionals (athletes and musicians) who are compared with amateurs; again contradictory results have been reported in terms of MRCP amplitudes (Fattapposta et al., 1996; Kita et al., 2001; Slobounov et al., 2002; Di Russo et al., 2005; Hatta et al., 2009; Wright et al., 2012b).

The event-related desynchronization (ERD) and event-related synchronization (ERS) are other EEG measures associated with movement (Pfurtscheller and Da Silva, 1999; Meirovitch et al., 2015). The ERD is a frequency specific power decrease that is often seen in the alpha/mu (8–13 Hz) and beta (13–30 Hz) frequency bands. The power decrease starts around 1.5–2 s before the movement starts, and continues until the movement onset. Up to 3 s after the movement has been terminated there is an increased synchronization primarily in the beta band (Pfurtscheller and Da Silva, 1999). The MRCP and ERD differ in terms of activation pattern and frequency and it has been suggested that they are generated through different neural mechanisms, although the same neural generators are active in both (Shibasaki and Hallett, 2006). As with the MRCP, the ERD has been shown to change as result of changes in neural plasticity and motor learning. It has been suggested that the magnitude of the ERD in the alpha/mu and beta band reflects the level of cortical excitability, with increases in excitability being associated with increases in ERD magnitude (Rau et al., 2003; Matsumoto et al., 2010; Noh et al., 2012; Aono et al., 2013; Kasuga et al., 2015). It has also been shown that a reduction in cortical excitability is associated with a reduction of the ERD amplitude (Cooper et al., 2008; Matsumoto et al., 2010). These findings have been consistent in terms of a larger or smaller ERD magnitude for increases and decreases in excitability, respectively, however the specific frequency ranges in which these changes are observed differs. For a single session of motor training, it has been found that the power decreases during and after the training (Andres et al., 1999; Serrien and Brown, 2003; Domingues et al., 2008; Nakano et al., 2013; Kiefer et al., 2014); however, the opposite has also been reported (Etnier et al., 1996). For studies where a task is trained over several weeks, the power has been shown to both decrease (Kaiser et al., 2014; Borghini et al., 2016a) and increase (Kerick et al., 2004; Borghini et al., 2016b; Houdayer et al., 2016). The affected EEG rhythms include theta, mu/alpha and beta, but again the reported frequency bands in which changes occur are not consistent across studies. The effect of long-term motor training on ERD has been investigated by comparing elite athletes with non-athletes. Decreased alpha ERD amplitude was observed for the athletes (Babiloni et al., 2009, 2010; Del Percio et al., 2009, 2010) alongside an increase in theta and alpha band power (Haufler et al., 2000; Baumeister et al., 2008).

Although the literature suggests that changes in movement preparation and execution are dependent on the phase of the motor learning, there is no consensus of how the MRCP and ERD are affected by single and multiple sessions of motor training. Therefore, the aims of this study were to investigate how the amplitude of the different segments of the MRCP and the ERD/ERS magnitude in the alpha and beta band are affected by motor training of a highly skilled hand motor task (laparoscopic surgery simulation) after (1) a single session and (2) multiple sessions using a limited number of EEG electrodesof motor training. It was hypothesized that the motor performance will be enhanced throughout the motor learning process with increased MRCP amplitudes and ERD magnitudes following the short-term motor training and a reduced amplitude/magnitude after the multiple sessions of motor training. One of the challenges in this study was the selection of a standardized task, to elicit the MRCP and ERD/ERS for quantification of changes following single and multiple training sessions. Since it is difficult to control the activation pattern, levels of force, and speed of the trained complex hand movements during laparoscopic surgery, a simple palmar hand grasp was used to elicit the MRCP and ERD/ERS instead. A palmar grasp is a movement that resembles the complex movements in the training task and can be standardized to elicit MRCP and ERD/ERS for quantification of changes with few electrodes over the motor cortex. We hypothesized that a difference in the MRCP and ERD/ERS associated with training of a complex hand movement can be quantified using a simple motor task (palmar grasp) that is different from the trained movements. To check this a control experiment was performed to investigate (third aim) if MRCP and ERD/ERS elicited from palmar grasps can be used to quantify training of another standardized hand movement (pinch grasp during a tracking task) (Falvo et al., 2010) to mitigate the effect of different tasks in quantification and training.

## Methods

### Subjects

Forty-seven healthy subjects were recruited in total and divided into groups according to the three objectives outlined in the aim. They were all naïve to the task. Sixteen healthy subjects were recruited for a single session of complex motor training; eight women and eight men (31 ± 9 years old). A further 18 healthy subjects were divided into two groups of nine subjects each. One group trained in six sessions over 2 weeks (five women and four men: 26 ± 12 years old), and the other group served as control (five women and four men: 29 ± 8 years old). Lastly, 13 subjects were recruited for the control experiment (nine women and four men: 21 ± 3 years old) investigating if a specific movement type could be quantified using another movement type than the one that was trained. The subjects' preferred hand was selected as the dominant hand; five subjects were left-handed in total. All the participants gave informed written consent and all procedures were approved by the local ethical committee of North Jutland, Denmark (N-20130081) in accordance with the Declaration of Helsinki.

The subjects were seated in a comfortable arm chair with their non-dominant hand resting on a table in front of them. Initially, the maximum voluntary contraction (MVC) was determined for a palmar grasp with the non-dominant hand.

### Pre-/post-training measurement

**Figure 1:**
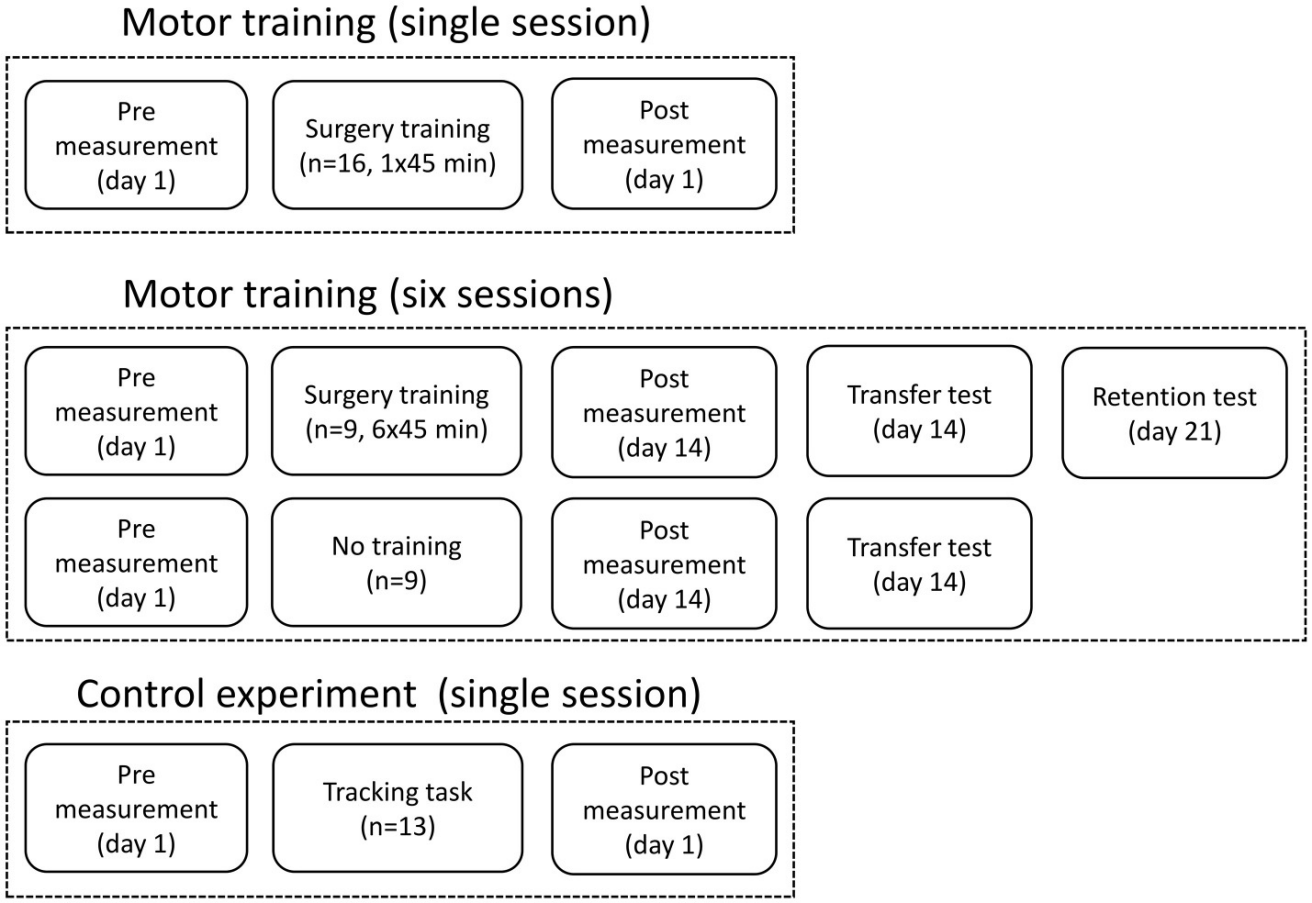
The figure shows the experimental design and when the recordings were taken and the how training sessions were distributed.

**Figure 2:**
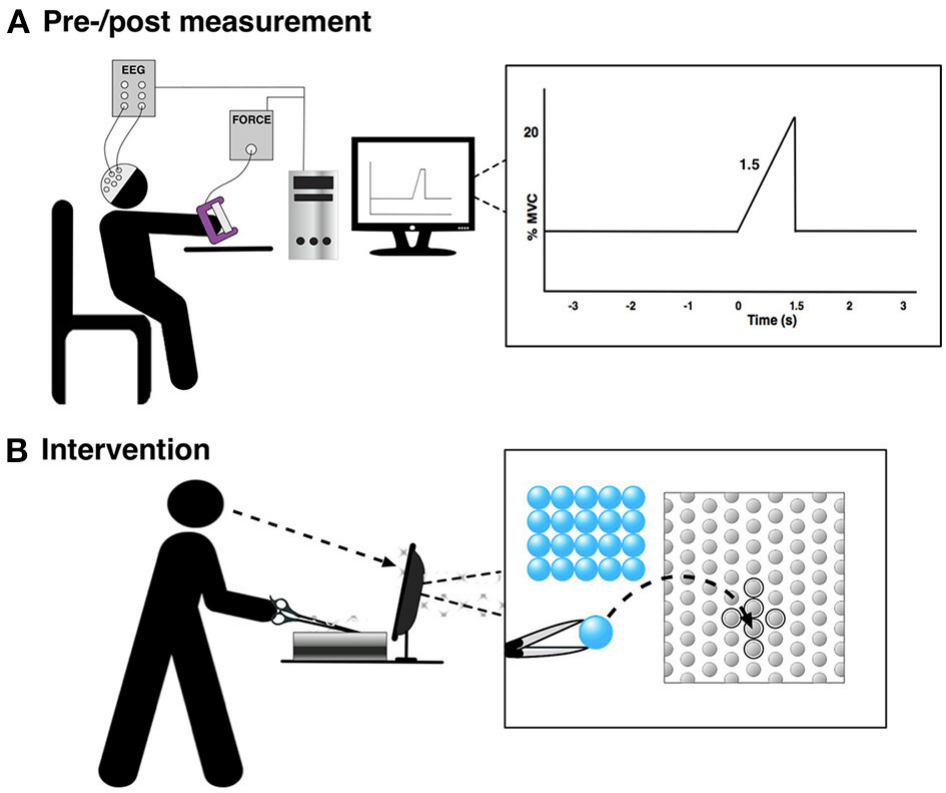
The figure shows the experimental setup for the pre-/post-training measurement and the training. “0 s” is the movement onset. **(A)** Pre-/post measurement and **(B)** Intervention.

The subjects were asked to perform 30 visually cued palmar grasps with their non-dominant hand in three sets each separated by a 10-min break. These were performed before and after the training (see Figure 1). The subjects were instructed to prepare to move for 3 s and then reach 20% MVC in 1.5 s (see Figure 2A). Each movement was separated by about 10 s. Grasp force was recorded and used as input to the custom made visual cueing program (Aalborg University), in which the subjects had continuous visual feedback while they tried to match the force template (Figure 2A). This was to ensure that the movements were performed in the same way throughout the experiment. Subjects spent 5 min familiarizing themselves with the task before the recordings were made. They were instructed to minimize blinks and facial EMG activity until after the movement was performed.

### Motor training (single session)

The training was 45 min of a difficult fine motor control task; laparoscopic surgery training using a simulator (NordSim, Aalborg University Hospital). The task was to pick up nine plate beads in total and put them in a cross on a plate using only laparoscopic forceps operated with the non-dominant hand (see Figure 2B). The subjects were unable to see the beads and the plate directly, but they could watch the forceps, beads and plate on a monitor. The subjects were instructed to complete as many patterns as possible in 45 min. The time to complete each pattern was noted.

### Motor training (six sessions) and control

The same training and measurements were performed as described above, but each subject in the training group trained 45 min 6 times over 14 days. The time to complete each pattern was noted in each session. There was at least a 24-h break between each training session. The pre-training measurement was performed at day 1 before the first session, and the post-training measurement was performed at day 14 after the 6th session. After the post-training measurement, the subjects had a break of ~30 min before they did another test, where 16 beads were placed in a 4 × 4 square on the plate, to investigate if they could transfer the learned skill to a novel bead plate pattern. The control group also completed this task, so the completion time could be compared between the two groups. The control group participated only in the pre- and post-training measurements with a 14-days break between. At day 21, the training group completed a retention test where 16 beads were placed in a 4 × 4 square again; the completion time was noted.

### Control experiment

A control experiment was performed in 13 subjects to investigate if a different movement, compared to what was trained, can be used to indicate changes in the EEG. This is tested since a palmar grasp (fairly simple movement) is used to elicit movement-related changes in the EEG instead of a more similar movement to what was trained (complex movement), which would be difficult to control and perform uniformly across subjects. The force tracking of randomly generated force templates was trained 50 times using a pinch grasp of the non-dominant hand. Before and after the training of the pinch grasp, 50 palmar grasps were performed with the non-dominant hand as summarized in Figure 2A.

### Recordings

#### EEG

Continuous monopolar EEG (EEG amplifiers, Nuamps Express, Neuroscan) was recorded using Ag/AgCl ring electrodes from FCz, C3, Cz, and C4 to cover the major cortical areas associated with movement preparation and execution of palmar grasp; the pre-motor cortex, supplementary motor area and primary motor cortex (Pfurtscheller and Da Silva, 1999; Shibasaki and Hallett, 2006). Electrooculography (EOG) was recorded from FP1. In the following, C4 and C3 for those that performed the movements with their left and right hand, respectively, will be called “contralateral channel” (to the non-dominant hand); the opposite channel, C3 and C4, will be called “ipsilateral channel.” The signals were sampled with 500 Hz and grounded at nasion and referenced to the right earlobe. The electrode impedance was checked before each of the recording lots, and the impedance was below 5 kΩ throughout the experiment. At the beginning of each of the trials (at “−3 s” in Figure 2A) a digital trigger was sent to the EEG amplifier from the visual cueing program to synchronize the EEG into epochs from the continuous recording.

For the multiple sessions motor training part, a different amplifier was used (g.HIamp, g.tec, Austria) but the same channels were recorded.

#### Force and maximum voluntary contraction

Force was recorded using a handheld handgrip dynamometer (Noraxon USA, Scottsdale, Arizona) and used as input to the visual feedback program to provide feedback to the subjects. At the beginning of the experiment three MVCs were performed. Each MVC was separated by 60 s, and the highest of the three peak values was defined as the MVC, which was used as the reference in the rest of the experiment.

### Data analysis

The EEG signals were bandpass filtered in different ways; from 0.05–5 Hz (MRCP), 8–13 Hz (alpha/mu), and 13–30 Hz (beta) using fourth order zero-phase shift Butterworth filters. All EEG signals were divided into epochs from 4 s prior the movement onset to 5 s after. Epochs were rejected if they contained: (1) EOG activity above 125 μV (peak-peak amplitude), (2) EEG amplitude differences above 100 μV, or (3) a positive gradient of a first order polynomial fitted to the data from the movement onset and 2 s prior this point (for the MRCP analysis).

From the cleaned epochs three types of characteristics were analyzed; segments of the MRCP and the ERD/ERS (alpha/mu and beta). Three segments were extracted from the MRCP from the averaged pre-training and post-training epochs: (1) the mean amplitude of the readiness potential from the beginning of the epoch until the start of the negative slope, (2) the mean amplitude of the negative slope in the interval from peak negativity and 500 ms prior this point, and (3) the peak amplitude of negativity corresponding to the motor potential. For the ERD/ERS analysis the same approach was used as in Pfurtscheller and Da Silva (1999). Initially, the EEG signals were bandpass filtered, and then the values were squared and averaged across trials followed by a 200 ms second smoothing window. The ERD/ERS was calculated in percent using the formula:

$$\begin{array}{l} ERD/ERS\;\left(\%\right)=100{\;}^{*}\;\left(A-R\right)/R \end{array}$$

**Figure 3:**
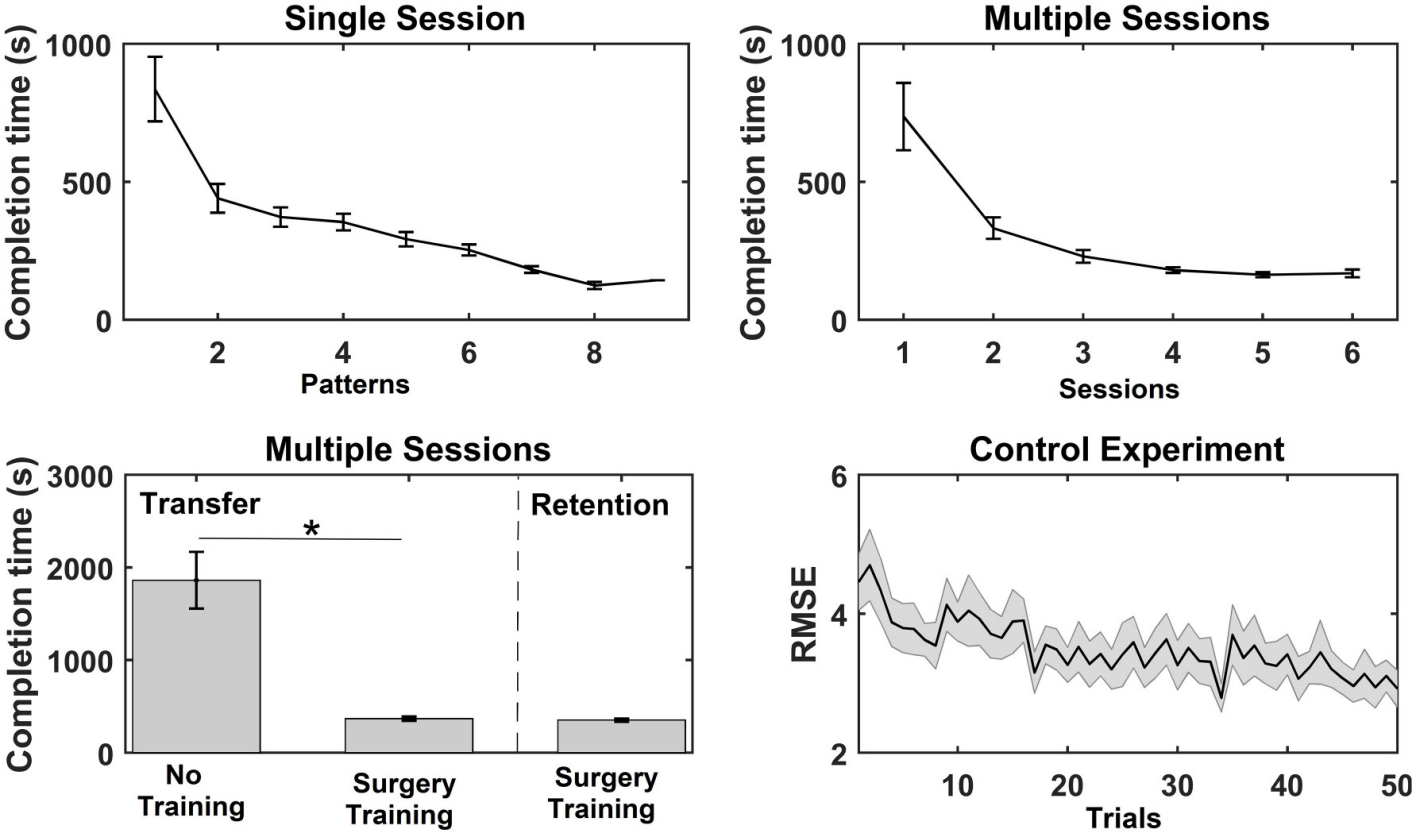
Overview of the results from the functional measures; completion time and root mean square error (RMSE) for the three experiments. The values are mean ± standard error. For the single session motor training, the average time to complete each bead plate pattern is shown (note that the number of completed patterns varied across subjects). For the multiple sessions motor training, the average completion time per bead plate pattern is shown for each training session, and the time to complete the transfer test for the control (Con) and training (Int) group and time to complete the retention test is reported. Lastly, the RMSE is reported for each trial in the training session (pinch grasp) in the control experiment. A significant test statistic is marked with “^*^”.

The reference period (*R*) was −4 to −3 s prior the movement onset. The activity period (*A*) was defined in three analysis windows: (1) the 2 s immediately prior the movement onset, (2) 0 to 1.5 s after the movement onset (execution of the movement, see Figure 2A), and (3) 1.5 to 5 s after the movement onset (ERS). The EEG characteristics were extracted from each of the four channels, which have been shown to capture the outlined EEG measures (Pfurtscheller and Da Silva, 1999; Shibasaki and Hallett, 2006). Two new channels were defined; contralateral (to the movement—i.e., C4 for left hand movements) and ipsilateral.

### Statistics

Initially, the functional measures were evaluated; completion time and root mean square error (RMSE) between the produced force and force template. For the single session motor training part, a Wilcoxon signed rank test was performed on the completion time of the first and last completed pattern. To evaluate if the completion time per pattern decreased throughout the six training sessions, a one-way repeated measures analysis of variance was performed with session as factor (six levels). The Greenhouse–Geisser correction was applied to account for sphericity violation. A *post-hoc* test with Bonferroni correction was applied. Moreover, the transfer test was compared between the control group and training group with a Mann-Whitney test, and the retention test and transfer test for the training group were compared with a Wilcoxon signed ranked test. To test if there was a reduction in the RMSE, the first half of the training trials were compared with the last half of the trials using a paired *t*-test.

In the single session motor training part of the study, the changes in each of the extracted EEG characteristics were compared for each of the four channels: FCz, Cz, contralateral, and ipsilateral electrode, from pre-training to post-training measurement. Each comparison was evaluated with a paired *t*-test, but a Wilcoxon signed rank test was used if the assumption of normality was violated.

For the multiple sessions motor training part, the change from day 1 to 14 was compared for the same EEG measures with a *t*-test or Mann–Whitney test if the assumption of normality was violated. Moreover, a paired *t*-test was performed for the average time to complete bead plate patterns on day 1 and 14, as well as a paired *t*-test to compare the transfer and retention test for the training group. A *t*-test was performed on the transfer test between the control and training group. The same tests were performed, as for the single session motor training part, when a palmar grasp was trained and quantified with a pinch grasp (control experiment).

The significance level was set to 5% for all tests.

## Results

**Figure 4:**
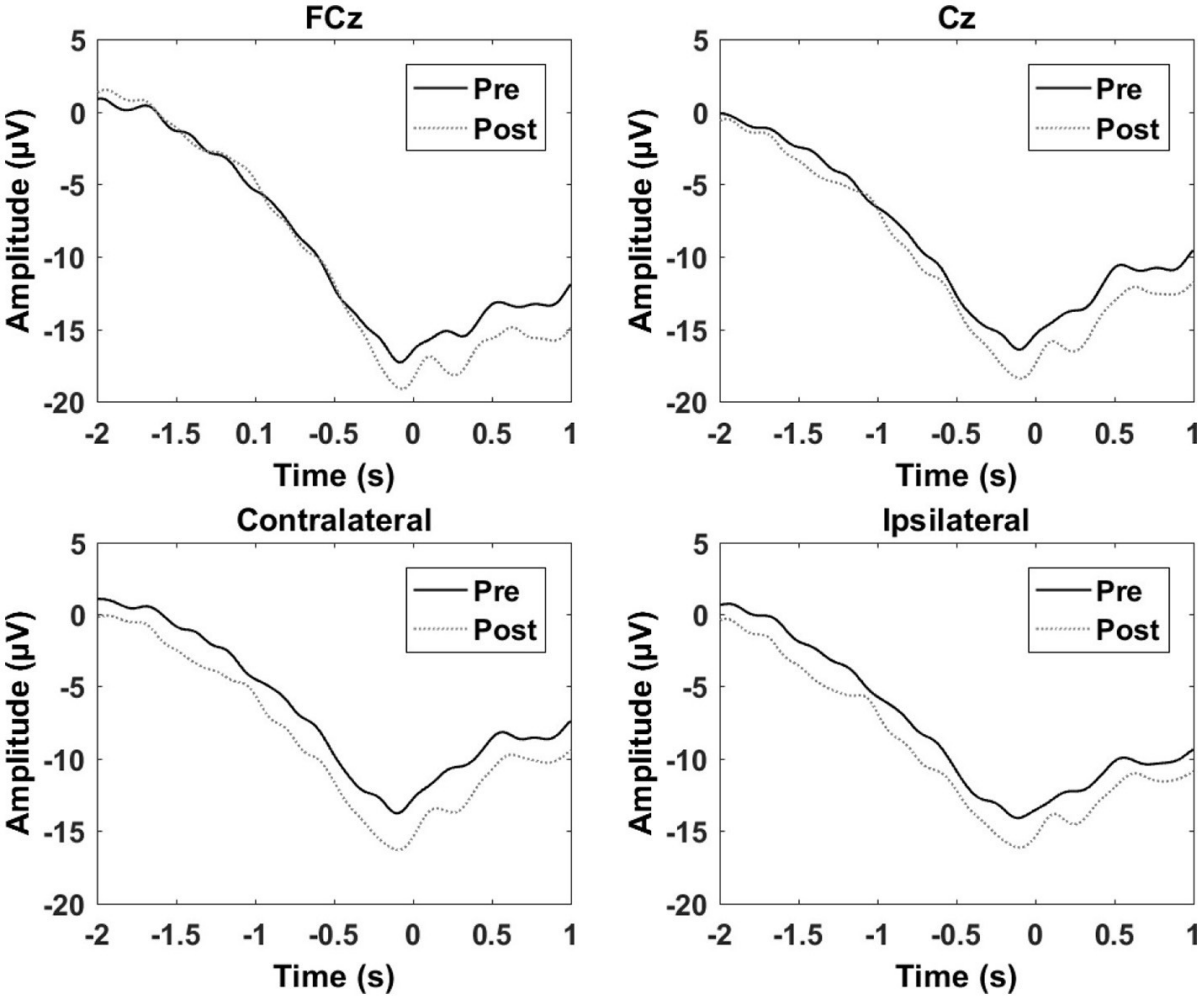
Grand average of the epochs across subjects for the motor training over a single session. “0 s” is the movement onset.

**Figure 5:**
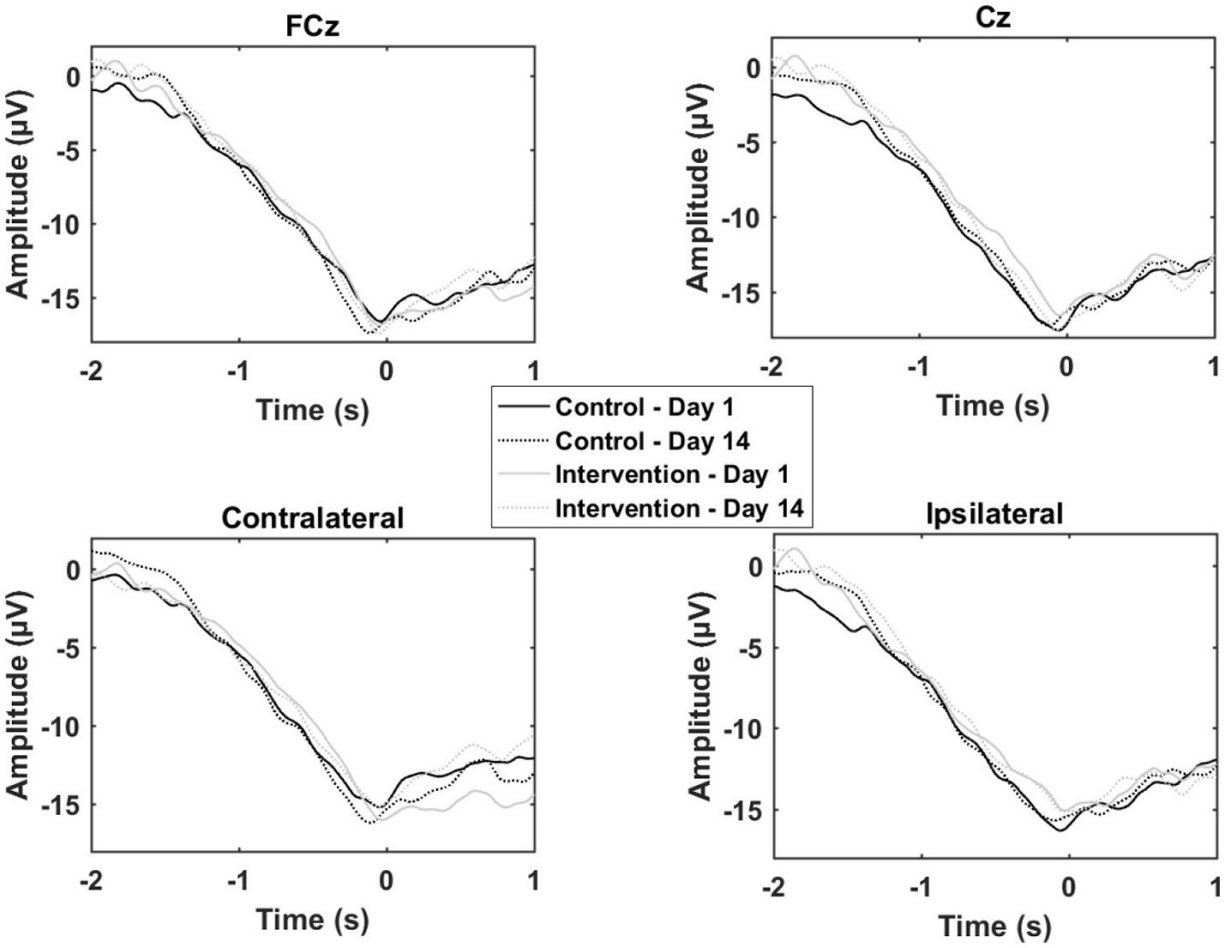
Grand average of the epochs across subjects for the motor training over multiple sessions. “0 s” is the movement onset.

**Figure 6:**
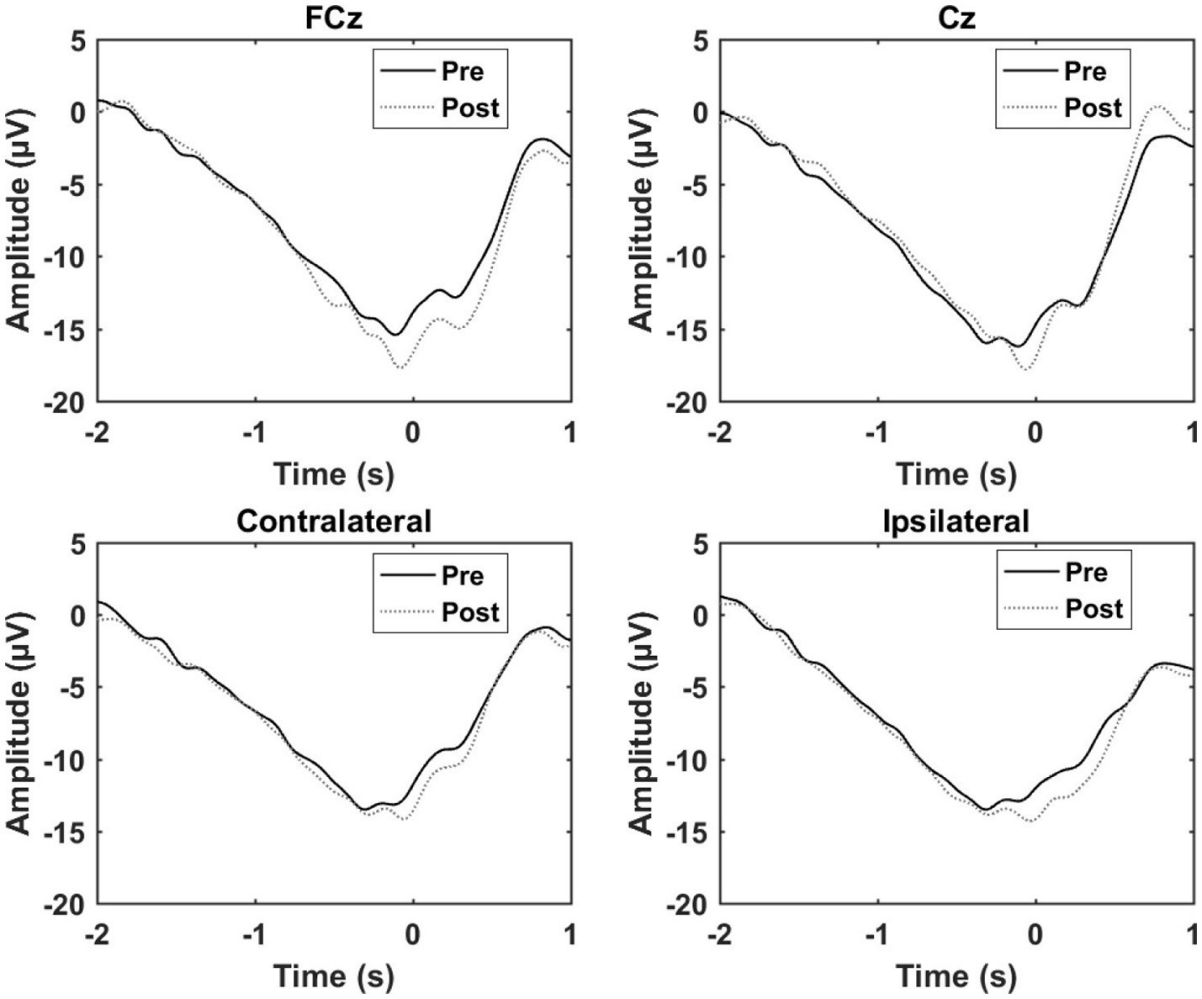
Grand average of the epochs across subject for the control experiment. “0 s” is the movement onset.

The results are summarized in Tables 1–**4** and Figures 3–**6**. Two subjects were excluded from the single session motor training analysis and one subject from the control experiment since all the EEG epochs in the post sessions were rejected due to the criteria mentioned above. On average 63 ± 36 epochs, 23 ± 10 epochs, and 13 ± 7 epochs were rejected per subject for the single session, multiple sessions motor training parts and control experiment, respectively. The sections have the following structure: single session training, six sessions of motor training, and control experiment.

**Table 1:** Amplitude differences (single training session) from pre- to post-training measurements for the different MRCP segments.

**Post-Pre (μV)**	**RP**	**NS**	**MP**
FCz	0.02 ± 0.7	−1.5 ± 0.8	−1.7 ± 0.9
Cz	−0.7 ± 0.8	−1.6 ± 1.0	−1.9 ± 1.0
Cl	−1.6 ± 0.7	−2.3 ± 0.9^*^	−2.6 ± 1.0^*^
Il	−1.7 ± 0.7^*^	−1.6 ± 1.0	−2.0 ± 1.0

“Cl,” Contralateral; “Il,” Ipsilateral; “RP,” readiness potential; “NS,” negative slope; “MP,” motor potential. All values are presented as mean ± standard error. A significant test statistic is marked with “^*^”.

### Functional measure

For the single session motor training, time to complete bead plate pattern was reduced over the course of the session. A Wilcoxon signed rank test on each subject's first and last bead plate pattern showed that there was a significant decrease in completion time from 836 ± 117 to 305 ± 46 s. A similar trend was observed for the multiple sessions motor training where the average completion time per bead plate pattern decreased significantly over the experimental sessions (see Figure 3). The *post-hoc* analysis revealed that the completion time was significantly shorter in session 3–6 compared to session 1 and 2. A significant difference was found in the completion time of the transfer test between the control group (1,861 ± 306 s) and training group (367 ± 23 s). For the training group, no difference was found between the transfer test (367 ± 23 s) and retention test (351 ± 18 s). Lastly, the RMSE decreased significantly as well, when comparing the RMSE of the first 25 trials with the last 25 trials.

### Movement-related cortical potentials

The results from the single session motor training experiment are summarized in Table 1 and Figure 4. It can be seen that there is an increase in negativity after the training. A significant increase was observed for the readiness potential for the ipsilateral electrode and for the negative slope and motor potential for the contralateral electrode. The greatest differences from pre- to post-training measurements are observed for the contralateral electrode.

The results from the multiple sessions motor training experiment are summarized in Table 2 and Figure 5. In general, the changes from Day 1 to 14 were similar for the control and the training group except for the contralateral channel. When comparing the control group with the training group a significant reduction was observed for the negative slope and motor potential.

**Table 2:** Average amplitude differences (multiple training sessions) from day 1 (D1) to day 14 (D14) in the different MRCP segments of surgery training and no training group and difference between the no training and surgery training group across all subjects.

**D14-D1 (μV)**	**RP**	**NS**	**MP**
**SURGERY TRAINING**			
FCz	1.9 ± 1.2	0.2 ± 0.9	−0.3 ± 1.2
Cz	−0.5 ± 0.7	−1.4 ± 1.0	−0.9 ± 1.0
Cl	1.4 ± 1.2	2.1 ± 1.9	2.1 ± 1.6
Il	0.2 ± 1.0	0.4 ± 1.0	−0.1 ± 1.0
**NO TRAINING**			
FCz	1.6 ± 0.9	−0.1 ± 0.7	−0.8 ± 0.8
Cz	1.2 ± 0.5	0.5 ± 0.4	−0.1 ± 0.4
Cl	−1.0 ± 1.6	−1.6 ± 0.4	−1.4 ± 0.6
Il	−0.4 ± 1.4	0.1 ± 0.9	−0.1 ± 0.7
**NO TRAINING—SURGERY TRAINING (μV)**			
FCz	−0.3 ± 1.0	−0.3 ± 0.8	−0.5 ± 1.0
Cz	1.7 ± 0.6	1.9 ± 0.7	0.8 ± 0.7
Cl	−2.4 ± 1.4	−3.7 ± 1.1^*^	−3.5 ± 1.1^*^
Il	−0.7 ± 1.2	−0.3 ± 0.9	−0.1 ± 0.9

“Cl,” Contralateral; “Il,” Ipsilateral; “RP,” readiness potential; “NS,” negative slope; “MP,” motor potential. All values are presented as mean ± standard error. A significant test statistic is marked with “^*^”.

The results from the control experiment are summarized in Table 3 and Figure 6. An increase in the motor potential was seen from the pre- to post-training measurement for FCz and the contralateral channel.

**Table 3:** Amplitude differences (control experiment) from pre- to post-training measurements for the different MRCP segments.

**Post-Pre (μV)**	**RP**	**NS**	**MP**
FCz	−0.3 ± 0.7	−1.4 ± 0.7	−1.9 ± 0.8^*^
Cz	−0.4 ± 1.0	−0.4 ± 0.9	−1.2 ± 1.1
Cl	−1.4 ± 0.8	−1.4 ± 0.8	−2.1 ± 0.8^*^
Il	−0.2 ± 0.4	−0.5 ± 0.8	−1.2 ± 0.8

“Cl,” Contralateral; “Il,” Ipsilateral; “RP,” readiness potential; “NS,” negative slope; “MP,” motor potential. All values are presented as mean ± standard error. A significant test statistic is marked with “^*^”.

### Event-related desynchronization/synchronization

The results for the ERD/ERS analysis are presented in Table 4. No changes were observed in the alpha ERD, but a significant increase in the beta ERD magnitude (the post-measurement recordings become more negative) of 6–7% points was observed for three channels; FCz, Cz, and the contralateral channel for the single session motor training. This change was observed during the movement preparation (T1) and movement execution (T2). For the multiple sessions motor training experiment there were no differences between the two groups. Moreover, no differences were observed in the control experiment. In general, the standard errors indicate that there is a fairly large inter-subject variability for all three experiments. ERD/ERS values exceeding 250% change were considered as outliers. One channel from the Alpha/Beta ERS analysis was removed from two subjects in the multiple sessions experiment.

**Table 4:** Changes in ERD/ERS for a single session, multiple sessions and control experiment at three different times: (T1) before the movement, (T2) during the movement, and (T3) after the movement.

**SINGLE SESSION**						
**Post–Pre**	**T1 (Alpha)**	**T2 (Alpha)**	**T3 (Alpha)**	**T1 (Beta)**	**T2 (Beta)**	**T3 (Beta)**
FCz	−2 ± 3	−2 ± 4	−1 ± 4	−6 ± 2^*^	−9 ± 2^*^	−1 ± 3
Cz	0 ± 4	−4 ± 5	1 ± 5	−6 ± 2^*^	−8 ± 2^*^	−2 ± 3
Cl	0 ± 3	−6 ± 4	0 ± 5	−6 ± 2^*^	−9 ± 2^*^	−4 ± 4
Il	7 ± 6	−2 ± 4	13 ± 7	−3 ± 2	5 ± 2	−1 ± 3
**MULTIPLE SESSIONS (NO TRAINING)**						
**D14–D1**	**T1 (Alpha)**	**T2 (Alpha)**	**T3 (Alpha)**	**T1 (Beta)**	**T2 (Beta)**	**T3 (Beta)**
FCz	0 ± 5	2 ± 6	6 ± 2	−2 ± 5	5 ± 8	−10 ± 9
Cz	−6 ± 5	−5 ± 6	1 ± 5	−2 ± 4	3 ± 8	−10 ± 10
Cl	−8 ± 8	−4 ± 9	0 ± 6	−4 ± 6	2 ± 9	−7 ± 10
Il	−2 ± 8	−1 ± 6	1 ± 5	−5 ± 8	−2 ± 9	−16 ± 15
**MULTIPLE SESSIONS (TRAINING)**						
**D14–D1**	**T1 (Alpha)**	**T2 (Alpha)**	**T3 (Alpha)**	**T1 (Beta)**	**T2 (Beta)**	**T3 (Beta)**
FCz	6 ± 6	3 ± 8	11 ± 5	−1 ± 6	0 ± 2	8 ± 6
Cz	5 ± 6	4 ± 7	9 ± 5	−3 ± 7	0 ± 2	10 ± 4
Cl	1 ± 11	6 ± 6	16 ± 8	−14 ± 16	0 ± 4	15 ± 18
Il	2 ± 8	7 ± 6	9 ± 7	−2 ± 9	5 ± 2	14 ± 12
**OVER ALL DIFFERNCE IN MULTIPLE SESSIONS (NO TRAINING-TRAINING)**						
	**T1 (Alpha)**	**T2 (Alpha)**	**T3 (Alpha)**	**T1 (Beta)**	**T2 (Beta)**	**T3 (Beta)**
FCz	−6 ± 5	−1 ± 7	−6 ± 4	0 ± 5	4 ± 5	−18 ± 8
Cz	−11 ± 6	−9 ± 6	−8 ± 5	0 ± 6	3 ± 5	−20 ± 7
Cl	−10 ± 9	−11 ± 8	−16 ± 7	11 ± 11	2 ± 7	−21 ± 14
Il	−4 ± 7	−9 ± 6	−7 ± 6	−2 ± 8	−7 ± 6	−30 ± 13
**CONTROL EXPERIMENT**						
**Post–Pre**	**T1 (Alpha)**	**T2 (Alpha)**	**T3 (Alpha)**	**T1 (Beta)**	**T2 (Beta)**	**T3 (Beta)**
FCz	−1 ± 4	−2 ± 5	−4 ± 7	−3 ± 4	−4 ± 4	−5 ± 5
Cz	−7 ± 7	−10 ± 8	−11 ± 10	−11 ± 7	−10 ± 6	−12 ± 10
Cl	−2 ± 5	−5 ± 5	−1 ± 8	−6 ± 5	−4 ± 4	−4 ± 6
Il	7 ± 6	3 ± 6	−1 ± 8	−3 ± 5	−4 ± 4	−2 ± 7

The values (%) are presented as a mean and standard error across subjects for the four different channels. A significant test statistic is marked with “^*^”. “D1” is Day 1, and “D14” is Day 14. “Cl,” Contralateral; “Il,” Ipsilateral.

## Discussion

All subjects improved their completion time of the bead plate patterns as a result of the training. As a result of the single session motor training an increase in negativity was observed for the different segments of the MRCP post training, and the ERD in the beta band was stronger (more negative) as well. For the six sessions of motor training, a decrease in negative slope and motor potential was observed for the contralateral channel, while no changes were observed in the alpha/beta ERD/ERS.

### Short-term vs. long-term training induces different cortical changes

The results from the analysis of the MRCP segments showed that the negative slope and motor potential of the contralateral electrode increased in negativity as well as the readiness potential for the ipsilateral electrode after the single session of motor training. The increase in negativity has been observed in the majority of previous studies as outlined in the introduction. The increase in negativity could be due to an increase in cortical excitability, which has been shown after hand motor skill training where improvements in performance were observed (Pascual-Leone et al., 1995). This hypothesis is supported by previous studies that have shown that increases in MRCP amplitude reflect increased cortical excitability (Birbaumer et al., 1990; Sato et al., 2015). It should be noted, however, that it also has been shown that the MRCP amplitude is inversely correlated with the amplitude of the TMS-elicited motor evoked potential (Lu et al., 2009). The modulation of the amplitude of the negative slope and motor potential in the contralateral motor cortex was expected given the activation pattern of the MRCP. However, the modulation of the readiness potential in the ipsilateral cortex was not expected although it has been reported previously (Smith and Staines, 2006). A bilateral activation pattern has been hypothesized for the initial part of the MRCP, but if this was the case then it would have been expected to see a significant decrease in the contralateral motor cortex and over the supplementary motor area as well (Shibasaki and Hallett, 2006). For the multiple sessions motor training, the opposite was found; a decrease in amplitude. Both an increase and decrease in negativity has been reported previously, but a limited number of studies have investigated the effect of motor training over multiple sessions. The reduction in the amplitude over the contralateral electrode could be due to neural efficiency where less cortical effort is needed to perform the task (Wright et al., 2012a). It may be speculated that the skill has become more automated when learning occurs, which is indicated by the transfer and retention tests, and that sub-cortical structures become more active (Doyon and Benali, 2005).

The findings in the current study regarding the beta ERD are in agreement with previous studies that have found an increase of the magnitude of the ERD after a single session when the trained task is still difficult to perform but not when the task is simple. For multiple sessions of motor training, when it has become easier to perform the task, no changes were observed for the beta ERD. It has been suggested that the ERD is attenuated with learning (Engel and Fries, 2010; Pollok et al., 2014). Contrary to other studies, the alpha/mu ERD did not change. The reason for this discrepancy may be due to methodological differences between studies, such as experimental setup, but it is also likely due to the subject-dependent nature of ERD frequencies, which have been reported to exhibit great inter-subject variability. In this study we used broad frequency intervals (8–13 and 13–30 Hz) and time intervals that were averaged; thus transient narrow ERD/ERS frequency intervals of e.g., 1–2 Hz may have been reduced.

### Evaluation of motor training

The findings in this study suggest that a simple movement such as palmar grasp can be used to quantify acquisition of highly skilled hand movements that are more complex than the palmar grasp; this is also supported by the control experiment. However, it could be that a greater increase/decrease in the MRCP amplitude or ERD magnitude could have been observed if the pre- and post-training movements were similar to those that were trained, though it would be difficult to control complex movements for variations in the executed force and speed which would affect the MRCP (Jochumsen et al., 2013). Volume conduction, however, would be an issue and it is likely that the recorded activity would not differ much from each other whether the skilled or simple movements were performed.

### Limitations

To reduce the variability in this study several single-trial MRCPs were averaged, but to obtain a better estimate more movements could be performed, and thus more trials included. The downside of this approach is that the subject will spend more time on performing the movements. Approximately five movements were performed per minute. The pre-processing of the MRCP trials could also be optimized to reduce the number of rejected trials due to EOG and drift of the signals, which again would increase the number of trials that was included in the average.

In future studies, the experiment could be replicated with more recording sites to obtain a finer resolution of the underlying brain activity. In this scenario, EEG source localization could also be investigated to determine if the training would lead to changes in the contribution from different brain areas as well as cortical dynamics during motor preparation and execution could be investigated with joint-time frequency analysis. Moreover, TMS-elicited motor evoked potentials should be investigated and compared to EEG to validate the relationship between MRCP and ERD which have been associated with cortical excitability and cortical reorganization. Lastly, the functional measures (time) could be complemented with a kinematic analysis of potential changes in the movement patterns of the hand and arm during the motor training.

## Conclusion

The amplitude of the readiness potential, negative slope and motor potential of the MRCP increased after a single session of motor training, while the negative slope and motor potential decreased after multiple sessions of motor training. An increase in the beta ERD magnitude was observed for the single session motor training, but not after multiple training sessions. Lastly, it was shown that a simple movement can be used to quantify a more complex movement. These findings are relevant for motor training studies where EEG is used as a tool to quantify the neurophysiological changes. With a simple setup and a low number of electrodes, it could potentially be a tool for clinicians to indicate neuroplastic changes following motor rehabilitation interventions; however, these perspectives need to be investigated further.

## Author contributions

MJ, IN, NS, KA, and DT were involved in the design of the study. MJ, CR, and HR were involved in data collection. MJ, IN, and SC were involved in the analysis of the data. MJ, CR, HR, SC, NS, KA, DT, and IN were involved in article writing and reviewing. All authors read and approved the final manuscript.

### Conflict of interest statement

The authors declare that the research was conducted in the absence of any commercial or financial relationships that could be construed as a potential conflict of interest.
